# Feasibility of a Personal Neuromorphic Emulation

**DOI:** 10.3390/e26090759

**Published:** 2024-09-05

**Authors:** Don M. Tucker, Phan Luu

**Affiliations:** 1 The Brain Electrophysiological Laboratory Company, Eugene, OR 97403, USA; phan.luu@bel.company; 2 Department of Psychology, University of Oregon, Eugene, OR 97403, USA

**Keywords:** neuromorphic computation, personal entropy, neural development, mortal computing, active inference

## Abstract

The representation of intelligence is achieved by patterns of connections among neurons in brains and machines. Brains grow continuously, such that their patterns of connections develop through activity-dependent specification, with the continuing ontogenesis of individual experience. The theory of active inference proposes that the developmental organization of sentient systems reflects general processes of informatic self-evidencing, through the minimization of free energy. We interpret this theory to imply that the mind may be described in information terms that are not dependent on a specific physical substrate. At a certain level of complexity, self-evidencing of living (self-organizing) information systems becomes hierarchical and reentrant, such that effective consciousness emerges as the consequence of a good regulator. We propose that these principles imply that an adequate reconstruction of the computational dynamics of an individual human brain/mind is possible with sufficient neuromorphic computational emulation.

## 1. Introduction

This paper considers the feasibility of replicating the essential features of a person’s brain and mind in informatic, computable form that can allow the subjective self to be maintained indefinitely through a high-bandwidth neural interface, with artificial intelligence. This process may be described as a transition to metaform. Our goal is a critical thought experiment to anticipate what might be possible with the accelerating technological advances of the near future. Although these advances are not yet realized, we propose that a creative, yet critical, analysis may clarify what can be determined as clearly not feasible, allowing us to plan for what is possible. As always, a historical overview may be required to understand what the near future entails.

An obvious preparation for this advancement has been the realization that a remarkable approximation of human mental capacity can now be reconstructed through artificial neural networks. Previous computational models of mental function (perceiving, thinking, planning action), with traditional, symbolic (Von Neumann) digital architectures, have been famously incompetent at approximating human mental ability. In contrast, from the initial implementation of distributed neural networks [1] it became clear to careful observers that brain-like function could be approximated through distributed neural representation [2]. This became described as *connectionism* and the general form in current engineering practice is often described as *general AI*.

The early connectionist models exhibited the *stability–plasticity dilemma*, in which training new weights (learning) led to the disruption of existing weights (prior learning). This was initially seen as a fatal defect in connectionist models for general computational purposes [3]. However, it soon became clear that the stability–plasticity dilemma is an inherent property of human, as well as artificial, intelligence [2,4] and it must be accounted for in any complex neuromorphic model. This may be a demonstration case that the inherent properties of distributed representations hold equally in brains and machines.

Although the theoretical insights into the significance of connectionist models were understood by certain researchers early on [2,5,6], it was only with mundane advances in consumer technology, including widely available parallel computation (Graphics Processing Units for computer games) and large accessible digital datasets (cat videos on the internet), that the significance of artificial intelligence (AI) became widely recognized, both by most scientists and by the general public.

Thus, the evolution of AI to this point could appear to be as much a technological accident as a principled intellectual discipline. Nonetheless, it may be worthwhile to attempt to formulate even naive principles on how intelligence is achieved similarly in machines and brains. Such principles might help evaluate the permeability of these forms of mental representation.

In this paper, we will present the case for the conceptual feasibility of a personal neuromorphic emulation (PNE), forming an interface between the human brain and AI. The essential theoretical advancement has been Friston’s formulation of active inference, the process of human cognition expressed by the minimization of free energy in information theory [7]. Extending and likely taking liberties with Friston’s reasoning, we propose that the informatic formulation of biological intelligence as a non-equilibrium steady state of the organism implies that the human mind has an informatic form that is not dependent on the mortal weights of the biological computer (the brain), but could be reconstructed by a sufficiently articulate information appliance. Clearly, actually constructing an accurate PNE will be a daunting challenge, requiring historical levels of investment, unique achievements in terms of human creativity, and some measure of time. Nonetheless, we propose that an adequate PNE is now feasible in principle, so that now may be the time to entertain a cost–benefit analysis.

## 2. Formulating Principles on the Physical, Informatic Basis of Intelligence

Taking stock of the advances in understanding the physical basis of intelligence in brains and machines, we propose three general principles that pave the way for achieving an interface with AI and, therefore, a path to lasting subjective intelligence through a PNE. We then propose a hypothesis, namely that the consolidation of active inference during each night’s sleep and dreams will, over time, manifest the full connection weights in terms of the historical self, such that measurement of the consolidation process over many nights will fully capture adequate information representation of the personal self.

### 2.1. Overview of the Principles

The first principle of physical intelligence is that *the memory that allows concepts, now in machines as well as brains, is implemented via a distributed representation of connection weights among elementary processing neurons*. The connection weights reflect *associations*, literally the strength of the associations among simple nodes or neurons in a large network architecture. For both brains and artificial intelligence, these networks are typically *multi-level*, so that the information is not simply at one associational level, but is achieved with multiple levels that can organize abstracted meaning at higher levels, thereby achieving abstract concepts. From this principle of distributed representational architecture, it is now clear that human brains and machines operate based on similar mechanisms of information processing.

Two additional principles are important, one from neuropsychology and one from physics and information theory. For the second principle of physical intelligence, when we study how psychological function emerges from the activity of the human brain, we see that there is no separation of the mind from the brain. The mind is not just what the brain does, it is the brain [8]. More specifically, the functions of the mind emerge literally from the neurodevelopmental process, the growth of the brain over time. This can be described as the *principle that cognition is neural development* [9]. The mind, in its neural form, is constantly growing, constantly organizing its internal associations (concepts) in exact identity with the strengthening or weakening of the brain’s connections. Because the process of the mind is always developing, the constituent neural connections of the mind must also be dynamic, growing, and regulated during that growth. The neurodevelopmental process of mind is the physical substance of our psychological identities, which are also (in exact register) profoundly developmental, changing and growing over time [9]. Importantly, we argue that this principle of neurodevelopmental identity implies that the mind, and the process of subjective experience, can be reconstructed fully from an adequate neurodevelopmental process description in information terms.

There is an important corollary of the second principle, already well-known in terms of machines and brains, namely that during the dynamic growth of connections, plasticity is achieved at the expense of stability [2]. The dynamic development of learning in terms of distributed representations operates in the same generic connection/association space, such that new learning invariably challenges the old memory. In the phenomenology of mind, careful reflection may teach us to recognize the old self that is put at risk with each significant new learning experience [10].

The third principle of physical intelligence is important for understanding the fundamental nature of information and why the information processing of the mind is not restricted to its evolved biological form (brains). This is what we derive from Friston’s [7] work as the *principle of the informatic basis of organisms.* In physics, we observe that the processes of matter, whether physical or chemical, tend toward greater *entropy*, meaning that they tend to lose their complexity and release energy. Life seems to defy this fundamental rule of thermodynamics, maintaining its complexity through the metabolism of external energy sources, homeostasis, and growth [11].

Recent advances in the physics of information have provided a new perspective on the organization of information that defines a living organism as a non-equilibrium steady state (NESS), a self-organizing, developmental form of information complexity that manifests rather than defies the fundamental physics of entropy in terms of the minimization of free energy [7]. If so, it seems to us that the cognitive functioning of this NESS, the self-evidencing that emerges during its operation as a good regulator (modeling its world for better or worse in order to live), could then be fully specified in an information description that is not unique to minds, or brains, or even biological things. Rather, the physics of information may offer a functional description of the mind with the same terms that apply to physical, entropic systems. The implication is that the neurodevelopmental process of organizing intelligence is not restricted to the particular biological form of evolved brains, but could be extended to a sufficiently complex computer.

Finally, an important practical point is that we can build tools to make tools. We do not need to build the PNE by hand, but instead can rely on neuroscience-informed artificial intelligence (AI) for the coding of a durable PNE. AI tools are now well-proven and rapidly improving, such that well-trained LLMs can assist scientists and engineers in digesting and integrating today’s complex literature. We just need to train them to embody the essential neuromorphic principles, such as those that are now manifested in the massive amount of neuroscience literature, in order to construct an advanced PNE.

We next attempt to explain these three foundational principles in ways that clarify their interrelations. These principles share the need to overcome the ingrained tendencies we have to restrict our thinking to familiar levels of analysis, where the psychological realm is somehow floating above the biological, the biological is somehow alive in a way the physical is not, and the computing of information is an abstraction that parallels but does not quite touch the stuff of these familiar and separate domains. Instead, we will imagine that there is but one phenomenon of intelligence and that we can learn to abandon our familiar categories of experience to describe it and, perhaps, achieve it, with a computable information theory of active inference.

### 2.2. The Identity of Mind and Developing the Brain Anatomy

The first two principles of distributed representation and neurodevelopmental identity are closely related, the first describing a static, idealized state of distributed representation and, the second, describing the dynamic network necessary for functioning human intelligence. Putting them together, the enabling insight for recognizing the feasibility of a PNE is the identity of the mind with the brain’s dynamic, growing, anatomical architecture.

The initial recognition came from studies of learning in brain networks, which showed that the regulation of connection weights in terms of synaptic strengths during learning followed the same activity-dependent specification as the formation and maintenance of synapses and their neurons in embryological and fetal neural development [12]. This is never a static implementation; the brain’s functional anatomy is continually developing through embryogenesis, through fetal activity-dependent specification of neural connections, through the growth of learning in childhood and then continuing throughout life [13]. The evolutionary structure of embryology is an important fact that must guide efforts to understand and emulate the organization of the human brain. Once the neurodevelopmental identity of the brain and mind are recognized, it becomes clear that a theory of the mind must be a theory of neural development, with the hierarchical organization of the brain reflecting its evolutionary developmental origins [14,15]. The neurodevelopmental identity principle can be stated in phenomenological terms: *cognition is neural development, continued in the present moment* [9]. What we experience in consciousness is the ongoing neurodevelopmental process that is constructing our expectations of the near future. Which all too quickly, of course, becomes the present moment, which will then be captured, or not, during the ongoing consolidation of memory.

The connectional architecture of the human brain is now understood sufficiently that a first approximation can be achieved for any individual’s brain. An important advance was the anatomical characterization of primate cortical anatomy, described as the Structural Model [16]. Current anatomically correct, large-scale neuromorphic emulations are now providing insights for interpreting neurophysiological evidence [17], implying the functional equivalence of brain activity with reasonably complex neurocomputational emulation.

Furthermore, the nature of the processing unit replicated in each cortical column is being specified by the computational model of the canonical cortical microcolumn [18], allowing the regional anatomical differences in cortical columns (which are now well-characterized in humans) to provide predictive information on the processing characteristics of each cortical region and, thus, providing generic human cortical column emulations. Recognizing that neural emulations could achieve reasonable accuracy through simulating cortical columns, rather than multi-billions of individual neurons, simplifies the computational emulation problem significantly [19], with a computing anatomy that AI-enabled science could soon specify in adequate detail.

With the realization that the mind’s information is represented in the connectional anatomy of the cortex, supported by the vertical (evolved, developmental) integration of the neuraxis, and with the increasing insights on the architecture of these connections that yields cognitive function, we can see that each act of the mind is implemented through the developing synaptic differentiation and integration of the brain’s physical, anatomical connectivity. With this realization, major features of human neuropsychology can be read from the brain’s connectional anatomy [9,13,15,20,21,22].

Of course, the mind is embodied: we experience and think through bodily image schemas [10]. Therefore, personal neuromorphic emulation must be a bodily emulation as well, very likely being subjectively acceptable only when provided with a functional, body-based avatar that feels like us, more or less.

At first, the principle of the identity of the mind with brain anatomy and function may seem to imply that the mind must die when the brain dies [23]. This is the theory of mortal weights [24]. The connectional structure (weights) of such networks will dissolve when we die.

However, because the brain/mind is indeed physical and, therefore, fully defined by its informational (entropic) content, the next realization is the possibility of identifying the information architecture of the brain/mind in information theory with enough detail that it becomes computable. Once it does, it should be possible to achieve a durable neuroinformatic replication of the individual self in non-biological form.

The insight is that if the mind is identical to brain anatomy, then a sufficiently accurate computational reconstruction of the developing (non-equilibrium steady state) brain anatomy (preferably with the joint emulation of the essential bodily homeostatic controls and their essential subjectivity) can then manifest a reasonable approximation to the personal mind, the subjective process, and the experience of the self.

Fundamentally, the emerging insight into the neurodevelopmental basis of experience prepares us to cross this next level of analysis, to understand the identity of intelligence not just in terms of the biological basis of the mind, but with information theory, as described next.

### 2.3. The Informatic Basis of Organisms Is Computable

As stated above, a fundamental schism between our understanding of animate (living) and inanimate (dead) matter has been represented by the concept of entropy. The laws of physics, particularly thermodynamics, require that all physical interactions tend toward greater entropy, the loss of complexity of form and the release of free energy. In contrast, life appears to find ways to avoid entropy [11].

This distinction has implied that two different sciences are required for living and non-living things. This implication may be wrong, as shown by more recent interpretations of the physics of self-organizing (non-equilibrium steady state, thermodynamic) living systems.

These interpretations have approached the brain’s cognitive function in terms of physical principles of Bayesian mechanics that align closely with the changing thermodynamics of physical systems [25,26,27,28]. The implication is that the self-organization of living organisms is not a fundamental violation of entropy, but rather a level of the non-equilibrium steady state of complex systems (biological organisms) in which minimizing free energy allows the growth and development of the self-organized form [7]. This theoretical advancement can be described as the *principle of the informatic basis of organisms.*

The Bayesian formulations of physical systems are aligned precisely with principles of information theory, consistent with the intimate relationship between information and thermodynamics in modern physics [29]. The emerging realization is that the physical mechanisms of complex, developing (Bayesian) self-organizing systems, like the human brain, may have an adequate functional description in information form [7,30,31].

Now, our proposal is that a sufficiently accurate information model should be computable and not be restricted to a particular physical form. Therefore, reasoning from the theoretical work of Friston and associates, we conclude that as the Bayesian mechanics of non-equilibrium steady state systems (brains) are characterized in sufficient detail in the near future, a computational implementation of that detail should naturally emerge.

Of course, there may be biological computers that remain mortal, with their inherent knowledge limited to hardware weights. But, in that case, the information-based theoretical description is inadequate, incomplete. If it is restricted to mortal computers [23], the theory of active inference may only be computable in biological form (in people’s all too mortal brains).

However, the advances in understanding the biological brain are being aided considerably by computational simulations. Such that we are understanding key neurophysiological systems by building computational simulations of them [17,32]. Furthermore, important advances have been made in neuromorphic emulations incorporating spiking models, achieving impressive cognitive capacities, such as inferring rules during intelligence assessment tasks, such as Raven’s Progressive Matrices [33], or emulating the primate visual system in regard to color perception capabilities [34]. These spiking models are clearly neuromorphic, in contrast to most of the connectionist architectures used in current image and language AI models, and innovative spiking models are being implemented with increasingly efficient spiking hardware architectures [35]. These demonstrations imply that an information theory description could be made to be highly neuromorphic for the human brain, implementing the physical principles of neural development that we now know to be identical to the principles of cognitive and emotional development.

If so, then the emulation of a specific brain should be possible through building a replicant neuroanatomical architecture and training it to achieve the emotional and cognitive functionality of that person, with extensive support through neuromorphic AI. As we will emphasize below, this may be best achieved in the near term by successfully emulating the memory consolidation of the enduring self during the essential neurophysiological exercises that are part of the nightly stages of sleep.

The replicant neuroanatomical architecture, with a high degree of precision in emulating an individual’s brain, is an essential starting point. We emphasize that the general architecture of the human brain is now understood in ways that can be aligned with current theories of active inference [21], and structural and functional neuroimaging provides detail on individual human anatomy and function [36]. The detail required for a replicant neuroanatomical architecture will require several orders of magnitude improvement over these generic models. The current neuroscience literature is vast and rapidly growing. We can imagine the development of a specialized LLM AI trained on the current literature and linked to advanced neuronal modeling software/hardware [37], allowing the construction of a high-precision, individual, neuromorphic model.

A revolutionary advancement is promised by the work involving neuromorphic quantum computing. The unique physics and, thus, the unique information processing of quantum computers are poorly adapted to classical instruction sets. But, they may be aligned well with neuromorphic information operations [38]. These operations may be implemented in continuous, analog form, such that we can emulate the high-dimensional complexity of neuronal activity in analog operations (in fact emulating neural activity, and as measured by the neuroelectric field, the brain itself). Although these developments remain in the early stages, they hold promise of emulating the learned weights of a person’s brain with sufficient accuracy to reconstruct the functional self.

### 2.4. Inferring the Weights of a Mortal Computer

In discussing the computational and power requirements for large deep learning models, Hinton [39] emphasizes that the assumption of conventional computation, that the software is independent from the hardware, may not be necessary for alternative computing methods. These methods could achieve effective machine learning with lower power requirements by modifying the hardware weights (rather than the power-hungry memory writes of conventional digital computers). Hinton described such an approach as “mortal” computing, in that the weights, being hardware, cannot outlive the hardware.

Practically, the weights of large, conventional, deep learning models can be seen to become fixed in a similar sense, in that—although the model as a whole may be copied—the weights are not easily reconstructed without repeating massive, and even difficult to reproduce, training programs. To address this problem, Hinton, Vinyals, and Dean (2015) proposed a *distillation* approach, in which rigorously emulating a large model’s predictions allowed the conversion of that model from a large cumbersome physical form into a more compact, or a more specialist, form [40].

Ororbia and Friston have recently explored the implications of mortal computing within the framework of active inference [23]. In their analysis, the assumption is still that a mortal computer, like a person, is restricted to the lifespan of its hardware. Under the first principle of physical intelligence, that connections are the basis of intelligence in both ANNs and minds, replicating the connection weights from a person’s brain is faced with a similar problem as when replicating the complex models discussed by Hinton et al. [40]: synaptic weights are not easily exported from the brain with the currently conceivable technology.

We propose that a possible solution is suggested by the realization that the connection weights of the human brain are not static, but are constantly developing, consolidating memories through the ongoing exchange of information between limbic and neocortical networks [41]. The synaptic weights are of course important, but the effective information during the consolidation of experience is not in the form of static weights, but it is rather their flux during the non-equilibrium steady state that is continually revised, continually developing, during waking, through the process of active inference and, during sleep, through the consolidation of active inference.

The Bayesian mechanics of memory consolidation (reflecting the reciprocal dynamics of active inference: generative expectancy and error correction) are complexly interwoven in the cognition of waking consciousness. We may not be able to recognize them as separate operations. However, we propose that these operations may be separated and laid bare, revealed in their primordial forms, through the sequential neurophysiological mechanisms for consolidating experience during the different stages of sleep. Generative expectancy, and the implicit self-evidencing of the core historical self, appears to be maintained and reorganized each night by REM sleep. Error correction and the learning about new states of the world may be consolidated in non-REM (NREM) sleep. A sufficiently accurate modeling of this complementary, active process of daily and nightly self-organization [13], when cast in the appropriate evolutionary developmental model, could provide the basis for inferring the information process of a PNE.

The result may be the development of information theory of active inference to characterize an individual’s brain sufficiently that it would be computable in general (immortal) form. The challenge may be to recognize that the active inference of experience in humans is developmental and dynamic, not in terms of static weights, but related to neurophysiological processes of ongoing consolidation that represent information dynamically during the continuing flux of synaptic transmission, rather than in terms of static synaptic strength. Importantly, as discussed next, whereas the synaptic weights of the human brain are not readily measurable, the flux of synaptic transmission in the consolidation during sleep may be measurable, with a sufficient level of characterization of the neurophysiology of synaptic organization during sleep.

### 2.5. Hypothesis: Active Inference Is Consolidated in Sleep and Dreams

To understand such neural dynamics, we propose the following hypothesis: *The neurophysiological mechanisms of sleep consolidate memory and, thus, the self, through negotiating the stability–plasticity dilemma of neural development in ways that are complementary to the waking process of active inference.* In brief, NREM sleep consolidates new learning [42]. This is the evidence of the world that corrects the errors of personal predictions, but in a way that stabilizes the new information within the neural architecture, inevitably at the expense of the old self (existing connections). The unpredicted events of the day become the significant uncertainties that must be integrated in the mind through the slow oscillations and spindles of NREM sleep.

Mechanistically, the unexpected information of the day’s experience engenders anxiety and, thereby, takes priority in the dynamic consolidation process of ongoing neural development, specifically in NREM sleep [22]. Transitioning from rumination to enduring knowledge, the potentially significant information of the day becomes the uncertainty-driven plasticity, the new learning, of the network consolidation in NREM sleep.

The companion to NREM sleep is REM, the paradoxical sleep in which our brains are active in the vivid, emotional, and bizarre experiences of our dreams. Neurobiologically, REM is the primordial mechanism for consolidating self-organization, establishing the instinctual structure of the genome through actively exercising this structure through the endogenous neurodevelopmental challenges of REM dreams [13]. The connections/associations of the network in dream experiences may seem to be quasi-random, phenomenally bizarre events. Yet they are experienced by the primordial self, the implicit actor in the dream (you), who must attempt self-preservation in each highly unexpected, unpredictable, dream scenario. The effect is to exercise the self-preservation affordances of the implicit self, the anonymous narrator, who experiences each dream episode.

Examining the emergence of REM in the human fetus, we can infer that the Bayesian expectancy of the core self, the generative process of active inference, is instinctual, initially specified by the ontogenetic instructions of the genome. As a self-organizing process, the genomic instructions are only the starting point, such that uniquely individual patterns of neural associations take shape early on and then develop the Bayesian momentum of the self well before birth. We begin self-organizing our integral neural architecture of activity-dependent specification through REM dreams fairly early in fetal development, around 25 weeks gestational age, well before we have any postnatal experience to consolidate [13,31,43,44]. All we have at this early fetal stage is a nascent human self, organized by the instinctual motives endowed by the genome. With the core (largely subcortical) mechanisms of the primordial self, hierarchically integrated across rhomb-, mes-, di-, and telencephalic levels of the neuraxis, we are able to exercise and grow these mechanisms during the quasi-random experiences of fetal REM sleep in ways that allow each of us to self-organize an increasingly general, organismic self, ready for the first experiences of infancy.

This dominance of REM in self-organization continues in the newborn infant’s first days and weeks of experience, when 50% of the time is spent in REM sleep. Only later, in the first year of postnatal life, does NREM become sufficiently well-organized to contribute its unique counterpart to neural self-organization, and the consolidation of the newly mature explicit memory, during sleep [13]. Just as NREM sleep consolidates recent, explicit, external memory in the mature brain [45,46], it seems as if the maturation of NREM sleep, later in the first year of life, achieves the increasing differentiation of the information boundary (Markov blanket), separating the individuated self from the social world [13,47].

REM continues the exercise and, thus, continues the integration of the primordial self during each night’s dreams throughout life, as an ongoing counterpart to the disruption involving new learning caused by NREM consolidation of new external information. An important paradox is that this REM process, by strengthening the integral stability of the self in the face of (unpredictable dream) plasticity, will provide the basis for generative intelligence through primary process cognition, the implicit manifestation of organismic creativity [27,48]. The mechanism for achieving stability of the implicit self, apparently by challenging it with the unpredictable, bizarre experiences of dreams, becomes the generator of novel creativity [49].

Although the theoretical formulation of the Bayesian mechanics of sleep as the complement to the waking elements of active inference is still in development, the basics of memory consolidation can be summarized in a way that emphasizes the opportunity for accurately modeling a living brain. The creation of memory, the ongoing stuff of the self, requires the consolidation of daily experience within the architecture of our existing cerebral networks, the Bayesian priors of the self. We do not need to extract the fixed weights of the mortal computer; we can observe these connections in the complement of active inference each night, in the dynamic (temporally developing) neurophysiology of our sleep and dreams. As the old self copes with new dream experiences, it manifests the essential Bayesian mechanics of its non-equilibrium steady state, the primordial self. This becomes an essential opportunity to see the network dynamics of the self during each sleep cycle. Over many cycles, in many nights of sleep, monitoring and learning to predict the high-definition electrical fields of a person’s cortex may provide a reasonable characterization of the enduring developmental self. Only through the consolidation of memory, does the self continue.

The pattern of human sleep, with five, or so, NREM–REM cycles, each night, is unique, even among big primates [50], apparently supporting the extended neural plasticity and developmental neoteny of the long human juvenile period [13]. This pattern varies from extended NREM and brief REM intervals in early cycles, to brief NREM and extended REM cycles later in the night’s sleep, apparently reflecting a shift from consolidating recent memories during NREM (favoring plasticity) toward more general integration of the enduring self during REM (favoring stability). As a result, the consolidation dynamics that are most important to emulate during the construction of a full PNE may be those of the late night, reflecting engagement of the foundational weights of the historical, childhood self. To the extent that NREM integration of external events balances the ongoing REM exercise of the implicit, historical self, these alternative modes of connection weight re-organization may reflect the variational Bayesian mechanics of self-evidencing through adaptive experience that could then be manifest in an adequate (dynamically self-consolidating) PNE.

## 3. Observing and Emulating the Consolidation of Experience

Modeling the dynamic consolidation of connection weights in an individual brain may, thus, be approached through observing and predicting both waking behavior, such as the experiences of the day that are tracked by a virtual personal assistant, as well as the consolidation of those experiences through measuring the neurophysiology of the enduring connectional architecture of the self during sleep. A person’s behavior may be sampled through the extensive digital archives that each person generates and it may be collected dynamically during the day’s activities. A natural method would be monitoring/predicting the person’s daily experience and monitoring/modeling each night’s sleep by a virtual personal assistant. Electrophysiological measures provide an integral and mechanistic way of creating an interface between the personal brain and the behavioral and linguistic image of the self, gathered and organized by the virtual personal assistant.

Clearly, the first way of creating an interface with the virtual personal assistant will be through verbalization. An LLM can be trained to record interactions and model them sufficiently, to reconstruct the person’s behavior (words and brain waves) and, thus, recreate their intelligence, with the patient and extended help of the virtual personal assistant. Electrophysiological modeling of the person’s brain is now possible in high definition, continuously day and night. A massive dataset can be constructed from a single individual over a few short months.

**Figure 1:**
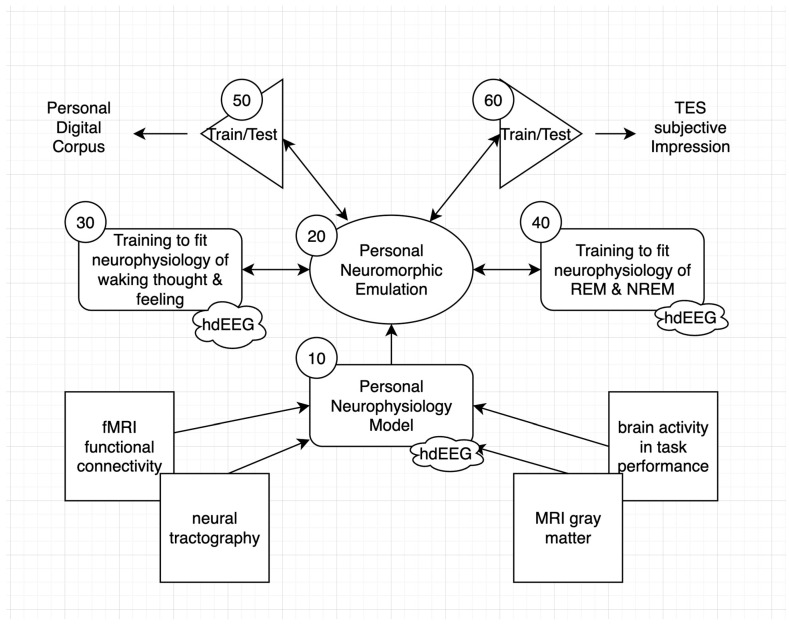
Elements of a PNE.

As outlined in Figure 1, the construction of a PNE (20) can begin by first reconstructing a personal neurophysiology model (PNM 10) from the individual’s anatomical and functional imaging (and thus a first approximation replicant neuroarchitecture), and then estimating the weights of this personal neural architecture by predicting the person’s ongoing cognition, emotion, and experience (30), in parallel with the physiological measures of brain activity (such as high-density EEG) assessed during these assessments. Constraints combining both function (behavior) and structure (the cortical anatomy in combination with neurophysiology) may be tighter and more productive than one of these aspects considered alone. As emphasized, the parallel collection of the neurophysiology during sleep may be essential to reveal the full connectional structure of the historical self, exercised, as proposed above, by adaptive coping in dreams (40).

The PNM can be constructed as a first-order architecture and, then, can be transformed through extensive training into a PNE that is built to be an enduring, self-regulating, informatic appliance of the self. Once the PNE is initialized and trained, it may be put to a validation test by predicting the individual’s behavior, as archived in a personal digital corpus (50). Because transcranial electrical stimulation (TES) has been shown to synchronize neural activity and alter ongoing neurophysiological rhythms [51], it may eventually prove possible to impress the PNE’s prediction of brain activity onto the person’s brain activity, to allow a subjective assessment of the accuracy of the prediction (a subjective Turing test).

### 3.1. Inferring Neural Connection Weights through Very High-Definition EEG

The measurement of the person’s neurophysiology, in terms of their waking behavior and experience and in sleep, may need to become more effective than is feasible with current methodologies, to ensure an accurate PNE. At the present time, the most important current neurophysiological measurement may be high-definition (also called high density) electroencephalography (hdEEG), defined as a head-surface (scalp, face, and neck) recording with sufficient sampling density (currently 280 electrodes, but additional information may be gained with up to 1000 electrodes). The hdEEG is a source localized to resolve the fields at the individual’s cortical surface, using a precise head conductivity model [52]. Because sleep is an essential aspect, it is important to be able to record the hdEEG during all the sleep during the night, as well as during daily activities (https://bel.company; accessed 1 September 2024). As understood within the theoretical model described above, the historical self is unlikely to be revealed fully during a few nights’ sleep, such that extended assessment, day and night, over months and even years, may be necessary, requiring a practical and comfortable assessment method.

Although it is often assumed that EEG has poor spatial resolution, considerable evidence now shows the ability to resolve cortical activity to a resolution of 1 sq cm, or so, on the cortical surface, with a 1 ms temporal resolution. Of course, higher resolution measurement may be necessary for accurate reconstruction of human neural activity in order to build an accurate PNE. Certainly, the obvious approach for capturing higher resolution data is to place recording (and stimulating) electrodes directly in the cortex for measuring local field potential (https://neuralink.com/, accessed 1 September 2024) (https://blackrockneurotech.com/, accessed 1 September 2024), with a corresponding loss of whole-brain coverage.

Recent unpublished work involving 280 hdEEG analysis with Multiple Sparse Bayesian priors, implemented using BEL’s Sourcerer software 1.0 [52,53], suggests that with the current hdEEG (now 280 channels), the tessellation of the cortical surface with up to 9600 dipole patches, yields well-structured source solutions across the cortical patches. With an estimated 200,000,000 cortical columns in the human cortex, this would provide an electrical field estimation for each patch for ~20,000 cortical columns. Certainly, there is local variation within these 20K columns that is lost to today’s hdEEG analysis and that may be significant to human brain function.

However, there is increasing ability to model the overall human brain columnar organization [32] within the Structural Model of the human brain [16] that can be aligned directly with the informatic analysis of active inference [18,21]. We propose that a PNM (Figure 1) can now be constructed, with a basis in the generic cortical column architecture of the human, aligned with the individual’s anatomy from neuroimaging (such as using Sourcerer’s Individual Head Model) and then trained to predict the cortical surface patches from extensive samples of the 280 channels in a hdEEG (for example, through a few years of day and night recordings, including extensive help and observation from the virtual personal assistant). The result would be a Bayesian super resolution from very high-density electroencephalography (vhdEEG) source analysis, wherein the prior model (initially the PNM and once it is trained, the PNE) incorporates the regularity of human cortical column anatomy to predict a very high-definition resolution cortical column activation model that can be trained initially with the lower-definition (9600 dipole) hdEEG source analysis currently available. For example, with the 10x super resolution achieved with this method, the source imaging would track the net activity of each patch from 2000 cortical columns.

The next step is to convert this PNM into a full, individual, behavior prediction model (the PNE) that is trained to emulate the individual’s full range of experience and behavior. In this example, a virtual personal assistant captures the person’s daily experiences and behavior in what may be called the personal digital corpus. The PNE retains the capacity to generate cortical electrical fields (and it includes subcortical networks integral to its architecture), such that synergistic dual constraints, fitting behavior, and fitting vhdEEG during sleep and awake become integral to the training. Because the science of TES is now rapidly advancing, such as in the ability to manipulate sleep neurophysiology [51], we can imagine that imposing very high-definition electrical fields on the cerebral cortex will generate subjective impressions that can provide further data for model validation.

### 3.2. A Personal Neuromorphic Interface to Evolving AI Architectures

There are, of course, obvious technical challenges to constraining the PNE to fit the person’s neurophysiological activity, as well as their behavior and experience, such that considerable expense and time will likely be required to find out what works. We think that even with considerable limitations in early working prototypes, a PNE could provide an effective two-way interface between the human brain/mind and neuromorphic AI architectures, suitable for creating a digital twin, such as now envisioned for medical, military, or industrial applications [54,55]. In order to emulate the actual brain, the PNE is limited in regard to one functional domain (the brain interface), namely to the architecture of the person’s brain. But, as an AI solution, it may also provide effective information exchanges with various AI resources.

An essential question for developing communication with the brain relates to the information process for memory consolidation. The information theory of active inference must be generalized beyond immediate learning to account for the ongoing neurophysiology of memory consolidation, through which both recent and historical experiences are continually woven within the architecture of the self [13]. The result will be an effective 24/7 interface between the brain/mind and evolving forms of AI.

Sleep promises to be the key to understanding memory consolidation and, thereby, unlocking the information organization within the brain’s computational architecture, during this process of creating an interface between the brain and neuromorphic AI. With increasingly high-resolution emulation of the personal neurophysiology of active inference in waking and in sleep, new forms of AI can be developed that will reveal the mechanisms of human memory consolidation and function. With these mechanisms revealed, they can then operate bi-directionally, allowing the AI to understand the mechanisms of a person’s experience and allow the person’s experience to incorporate the advanced memory structures of the personal neuromorphic AI.

Certainly, the assertion that adequate personal neuromorphic emulation can be constructed will only become convincing once actual progress is made, leaving the current proposal as little more than a thought experiment. However, the exercise to this point, allowing us to imagine the operation of a very high-definition, hybrid, brain–machine interface, does raise interesting issues. For example, even as the PNE is trained to mirror the operations of the person’s brain [56], this would not be an *exact* duplication, but would reflect the inherently unique self-organization of the PNE itself.

More fundamentally, the AI tools used to construct the PNE, and to create an interface between the PNE and the person’s brain in order to connect to AI resources, would almost certainly reflect transformational events, such that—rather than the simple transition of the person’s brain/mind to the informatic form that we envisioned at the beginning of this paper—the effective memory and consciousness of the brain/mind–PNE interface would create a new, hybridized brain–AI form of intelligence.

At this time, we can only wonder what this will feel like. We understand the familiar forms of subjective experience, more or less, but even the natural mechanisms of memory consolidation and, specifically, the exercise of these mechanisms during the nightly stages of sleep are largely opaque, even when subject to careful reflection. We might imagine that the reflective insights that a PNE could provide, insights into the mechanisms of experience and memory, might be incorporated within the subjective process. With insight into the mechanisms of memory, should come insight into the neural mechanisms of consciousness, and the hybrid forms that may ensue. Even though we started this exercise with the simple notion of self-replication in informatic form, it becomes clear in studying the necessary construction of a neuromorphic interface that, regardless of the specific form that hybrid, neural AI architectures will take, the transition to metaform will almost certainly present challenges to the experiential continuity of the self.

### 3.3. Phenomenology of the AI Interface: Is This Still Me?

Even if it feels like me, can I give up my embodied brain and the continuity of the old self I have known all these years for this hybrid informatic form? Or, as my body surely fails, should I just accept personal entropy and the quiet fade into oblivion, considering that the evidence of nightly sleep consolidation provides an instructive perspective on this question of the experienced continuity of the self?

Although most of us experiencing a reasonably stable mental status assume that we are continually aware of the continuity of the self, careful neurophysiological analysis raises questions about this assumption, showing that the continuity of the self is an illusion we construct to interpolate experience over the profoundly unconscious, and the occasional bizarre dream conscious, experiences that transform the self during each night’s sleep.

Whereas a logical philosophical position can easily state that the self is a stable fixed entity, such that a hybrid informatic fusion of our network weights with an advanced PNE would do irreparable violence to this entity, the neurodevelopmental process of sleep shows a more nuanced life of the enduring mind.

In order to consolidate new experiences (through NREM) without catastrophic interference, the self appears to exercise and reconstruct its enduring Bayesian priors in the phenomenal struggles of each night’s REM dreams. Because significant new information has been incorporated with the plasticity of new learning, and because the ongoing reconstruction of the stability of the self is approximate (because new information has been incorporated), it is then likely that we wake up each day in a somewhat new form, even as we naturally interpolate (hallucinate) the experience of a continuous self.

Given this variable continuity of the biological self, and despite our ability to experience the continuity of the self through each night’s transformation, we might find that as we gradually take the form of a reasonably accurate personal neuromorphic emulation, with AI hybridization, it may seem like just another day in life, not so different from waking up from our familiar sleep and dreams.

A particularly important practical reality of identifying with the PNE and its interface with the artificial realm is that, given the complexity of emulating a person’s neural activity, likely requiring unprecedented development of computational neuroscience AI tools specialized for this process, each person will likely experience various states of artificial intelligence enhancement before abandoning the (necessarily mortal) biological brain. The process is, therefore, likely to be a relatively gradual transition to hybridized form, or metaform, rather than an existentially abrupt displacement.

## 4. Consolidating Experience through Active Inference

In addition to technical and phenomenological questions of feasibility, the general scientific feasibility of a PNE–AI hybridization rests on the ability of the informatic description, of the active inference applied to the neurodevelopmental process, to account for the major qualities of the human personality. We want to be maintained in a reasonably whole form. The information theoretic characterization of self-organization through active inference provides abstract and general concepts for understanding the boundaries of the self and the process of self-evidencing [7,26,27]. The utility of this approach (and perhaps even the best argument for its validity) is seen in the insightful explanations that active inference provides in developing more specific formulations, such as in the neocortical mechanisms of expectant perception [18], or the role of implicit limbic–cortical predictions of anticipated feeling in guiding the control of action [28]. These more specific functional articulations support our conjecture that an information theory description will soon be extended to the full functioning of the human mind.

In support of this notion, we now point to several papers attempting to align the neurodevelopmental mechanisms of the personality with the process of active inference. Active inference is an ongoing process, in waking and sleep, that consolidates experience through neurophysiological mechanisms that can be observed and understood in real time, physically. In these several papers, we argue that the Bayesian predictive model of active inference can be extended to describe not only the generation and correction of representations in the cortex (cognition), but also the adaptive control systems of subcortical and limbic networks that underlie generative processes (primarily through the lemnothalamic regulation of dorsal corticolimbic communication) and critical, corrective processes (particularly through the collothalamic regulation of ventral corticolimbic communication). This theoretical framework may be consistent with the interpretation that active inference reflects control systems as much as representational mechanisms in the brain [14]. The important effect of recognizing differential motive control systems underlying active inference [49] is to extend the architecture for active inference from the cortex to the evolved, vertically-integrated hierarchy of the human neuraxis [15].

By extending Bayesian mechanics to the rich anatomical basis of multiple evolved levels of neural control systems, this theoretical work attempts to bring the full complexity of the biological brain into the generative developmental framework of active inference. Although the theory is only in its early stages, it forms the basis for our assertion that the phenomenal self and its essential psychological capacities of cognition, emotion, and personality, can be recognized and interfaced with AI through emulating the neurodevelopmental process of self-organization and ongoing memory consolidation through the informatic description of active inference.

### 4.1. Growing a Mind through Active Inference

The theory of active inference [7,57] provides a Bayesian computational model for maintaining complexity (minimizing free energy) that can be aligned with the connectional architecture of the human brain [18,28]. The Bayesian mechanics of this computational model inherently define the self within the boundary of the Markov blanket that differentiates the self from the environmental context [31,58].

To achieve a functional replication of the self, a PNE must be given the mechanisms for self-organizing in the same way that the person’s personality is self-organizing, through the ongoing consolidation of experience by the emotional and motivational processes that both vertically integrate the neuraxis and achieve individuation of the personality within the social context. Several recent theoretical efforts have attempted to clarify the neurodevelopmental processes of personality self-organization and self-regulation in ways that accord with the principles and mechanisms of active inference simultaneously with the principles of neuropsychological development. To the extent that these ideas are a reasonable approximation to the human neurodevelopmental process, they can serve as a first design for specifying the self-organizing capacities that must be included in the construction and development of a personal neuromorphic emulation.

An initial effort was to build upon the insight that cognition is the ongoing process of neural development, forming the neuronal connection weights that specify both the neurocomputational architecture and the structure of the personality [9]. A key realization in this approach is that animal (and human) learning proceeds first through motivated expectancies that anticipate hedonic affordances in the world (described as the *impetus* of elation and positive affect) and then these expectancies are differentiated by the negative feedback from the environment through a process of self-constraint (the *artus* of anxiety and uncertainty).

This theoretical formulation thus proposed that there are unique forms of emotional and motivational control that differ for the dorsal (spatial, configurational) versus the ventral (object, focused) forms of memory that are emergent from the corticolimbic dynamics in the mammalian brain [59]. The neuropsychology of personality can then be seen as a hierarchical integration of motive control (from limbic networks) with the cognitive representational capacity of the dorsal and ventral division of the neocortex [9]. In a series of papers, described in the next few sections, we suggest that the information theory of active inference provides an organizing framework for understanding corticolimbic dynamics in terms of emotion, cognition, and personality.

An important insight that emerged in this work is that neuropsychological development is inherently Bayesian: the personality, the historical self, is the unavoidable reference for each perception and action [60]. As the self generates expectancies for self-actualization (predictions) through the *impetus*, it is guided by the motive arousal of elation, leading to an extraverted mode of personality functioning, motivated by hedonic expectancies (primary process cognition). In a complementary fashion, the personality is also regulated by the ventral limbic control system, in a secondary process form of control through negative feedback and error correction from contacts with the world through the *artus,* the motive control exerted by anxiety and constraint [9,22].

### 4.2. The Limbic Base of Adaptive Bayes

To specify the neurocomputational mechanisms of the cerebral architecture that implement active inference in its full anatomical form, Tucker and Luu [21] reasoned how active inference must be implemented within the primate corticolimbic network architecture that defines the human brain’s connectional organization. In conventional neuroscience theory, the networks of the sensory and motor regions contacting the world are the source of “bottom-up” sensory data; these are thought to be regulated by “top-down” control from higher (more cognitive) cortical networks, typically considered to achieve the highest representation in the heteromodal association cortex. However, the connectional architecture detailed by the Structural Model [16,61] shows that the limbic cortex is still higher in the cortical hierarchy, providing the heteromodal cortex with limbifugal expectancies from limbic, motivational, and emotional regions. Just as the organization of memory is now understood to depend on the regulation by limbic networks applied to the entire corticolimbic hierarchy, the adaptive, motive control of active inference in personality must be understood to originate in the self-regulation emanating from the adaptive limbic base. Neurodevelopmental self-organization of the personality is thus achieved through the ongoing adaptive consolidation of experience, which anchors the neocortical construction of adaptive representations of the neocortex with the more fundamental motive control systems of the generative impulse (impetus) and anxious constraint (artus) emerging from the dorsal and ventral limbic divisions, respectively.

### 4.3. The Phenomenology of Active Inference

If PNE is to effectively reconstruct personal experience, it must resonate with the subjective consciousness in a way that is coherent with the experience of the self, while being in biological form. This might be described as the criterion of the subjective Turing test. The neurodevelopmental theory of cognition that identifies the mind’s capacities within the network architecture of the Structural Model must account for the multiple representational levels of the human cortex, including three levels (*layers* in machine learning terms) of the limbic cortex (classically described as the allocortex, periallocortex, proisocortex) and four levels of the neocortex (heteromodal association, unimodal association, primary sensory, motor). For more information, see Figures 3 and 6 in [22].

How does consciousness arise from active inference working across these multiple layers? Is there a component of phenomenal experience that can be identified with the limbic levels of representation, as separate from the neocortical level? Fundamentally, the strong feedforward mode of spatial, contextual cognition in the dorsal corticolimbic division (contributing to the impetus and associated with a minimal or even absent layer 4) would seem to bias experience in the limbifugal direction in terms of hedonic expectancies. In contrast, the elaboration of feedback control in the focused object cognition of the ventral corticolimbic division, associated with a well-developed granular layer 4, would appear important to the more articulated cognition organized from the highly developed error correction. Is there a phenomenology of consolidating experience that can be explained by these neurocomputational variations in Bayesian mechanics?

Tucker, Luu, and Johnson (2022) proposed that several classical phenomenologies, such as those by Peirce, Dewey, and James, have emphasized an implicit form of consciousness that may often be described as intuitive experience [62]. In contrast, the more explicit form of consciousness, typically emphasized as the “hard problem” of consciousness in modern philosophical [63] and neuroscience approaches [64], only emphasizes the articulate consciousness of the focused contents of working memory (the pristine qualia). If a PNE is to reconstruct the full range of subjectivity described by classical phenomenology, then the neurocomputational architecture must be sufficient to allow attention to shift from implicit (contextual and widely associated) consciousness to the more explicit (focused and narrowly associated) experiential forms more typically identified as consciousness.

### 4.4. The Differential Precision of Variational Bayesian Inference

The adaptation of the neurocomputational model of active inference in order to operate in terms of both dorsal (archicortical, contextual memory) and ventral (paleocortical, object memory) architectures may allow the PNE to reflect the functional connectivity networks now well-characterized by resting-state, functional, MRI connectivity networks. Tucker and Luu [22] present a theoretical analysis suggesting that the dorsal and ventral attentional systems in the current functional connectivity literature have cognitive properties (endogenous direction dorsally reflecting the impetus; stimulus-reactive direction ventrally reflecting the artus) that align not only with classical neuropsychology [59,65], but also with the findings in the fMRI functional connectivity literature [66]. This analysis thus suggests how the motive controls from dorsal and ventral limbic divisions can thus provide the personality, and by extension the PNE, with the unique forms of self-regulation that are integral to the familiar human attentional and cognitive systems.

These different forms of motive control can be seen to bias the process of active inference toward an emphasis on the creative, generative feedforward control of the dorsal cortical division, contrasting with the critical, evaluative feedback control of the ventral cortical division. The balance between weighing the prediction (feedforward expectancy) versus the evidence (feedback error correction) in Bayesian analysis is often attributed to judgments about the *precision* of the evidence. However, in the evolved architecture of the mammalian brain, the dorsal and ventral limbic divisions appear to reflect relatively parallel computational divisions, with differential balance toward weighing predictions (dorsal) versus evidence (ventral) in their processing architectures. This may suggest that, in the spirit of variational inference, certain networks come to the fore for creative generation (impetus), whereas, at other times, the mind is dominated by the networks for critical constraint by evidence (artus). The result is that active inference alternates in terms of variational inference as a dialectical (opponent and complementary) process [67]. The differential modes of this process are motivationally primed mechanisms for consolidating memory.

### 4.5. The Challenge of Neural Self-Regulation through Vertical Integration

The specification for computing organismic neural development is provided by the human genome, with the implementation of each individual coded by the unique phenotypical variations of primordial germ cells and embryogenesis. Beginning with the neurodevelopmental principles of active inference in human corticolimbic networks outlined above, an essential challenge is to specify the self-organizational process through which these corticolimbic dynamics, within the human telencephalon, are emergent from the more elemental dynamics of vertical integration across the evolved levels of the subcortical neuraxis. Understanding the hierarchical organization of control systems for active inference in the brain [14,57] may be facilitated by considering the Bayesian mechanics that are intrinsic to the rhombencephalic, mesencephalic, and diencephalic foundations that determine the dynamic and ongoing mechanics of the telencephalon [15].

Ontogeny, the computational organization of the individual, does not recapitulate phylogeny, the evolution of the species, exactly. Yet it reflects the developmental species residual of phylogeny, the elements of the species genome that that have been retained in human evolution to guide the ontogenetic, neurodevelopmental process of the mind for the specific person, initially, necessarily, along the general lines of phylogenetic organization. Certainly, there are powerful epigenetic mechanisms operating throughout the neurodevelopmental process, yet the evolved control systems provide a fundamental cybernetic hierarchy for motivating the dorsal and ventral limbic contributions to active inference. As a result, the contributions of the vertically integrated primitive levels of the brain are essential to characterize, at least in general form, and explain the ongoing self-organization of the individual neurodevelopmental process that defines the personal mind and its eventual continuance in the informatic form of PNE.

To understand the subcortical arousal and motivational control of the dual, dorsal and ventral, limbic–cortical systems, Luu et al. [15] used advances in modern neuroanatomy, informed particularly by the prosomeric model of evolutionary developmental analysis [68], to trace the anatomical roots of the dorsal and ventral corticolimbic divisions to their brainstem and midbrain control systems (phasic arousal and tonic activation, respectively). The key anatomical insight [69] was the recognition of dual paths of connectivity through the diencephalon, the thalamus, described as the *lemnothalamic* (connecting the brain stem lemniscus or ribbon-like fiber tracts) and *collothalamic* (connecting the midbrain colliculus with the telencephalon) paths.

The implication of this analysis is that the self-regulation of the individual’s telencephalic architecture in terms of the Bayesian mechanics of active inference emerges from unique and yet primitive forms of motive control, which have long been integral to the evolution of the vertebrate brain. The lemnothalamic projections from the lower brainstem (the classical reticular activating system) regulate the dorsal limbic control of the contextual impetus through a *habituation* bias, a unique cybernetic form associated with the mood of elation that facilitates generation of expectancies in the limbifugal direction. In contrast, the collothalamic projections from the collicular midbrain are particularly important to the ventral limbic division and striatum, regulating the tonic activation stabilizing actions through a redundancy or *sensitization* bias, an activation control with the subjective properties of anxiety that is uniquely suited to regulating constraint and error correction in the limbipetal direction of neocortical processing, in particular [15].

Although this evolved subcortical anatomy of human active inference is complex, it is highly conserved by evolution, such that a generic subcortical computation model may be adequate for starting the training of an individual PNE, with the recognition that unique personality features will be explained by the motive controls that are tuned to be consistent with the unique information processing modes of the dorsal and ventral limbic divisions [15].

### 4.6. Self-Regulation of Active Inference and the Predictable Consciousness of a Good Self-Regulator

Given that the goal of a PNE is to emulate the individual mind through an advanced informatic process, it may seem surprising that the theoretical work reviewed above focuses on characterizing the continuity of vertical integration of the neuraxis through fundamental vertebrate neural control systems. Is the evolutionary context of the human brain so important to the artifacts we design to succeed it?

Yes, most definitely. To capture its inherent architecture, a hybridization of the human mind with AI, indeed, requires emulation of the full hierarchy of the neuraxis that has allowed the human brain to regulate itself through the extensive modifications of primitive vertebrate control systems throughout evolution. Certainly, we can conceive of the next steps in the evolution of intelligence, where the success of PNEs allows us to experiment with various fusions of human and artificial intelligence, almost certainly requiring exotic computational advances, such as neuromorphic quantum computing. But if we are to replicate human intelligence, the first step must be a functional replication of the individual mind with the complex architecture of vertically integrated rhombencephalic, mesencephalic, diencephalic, and telencephalic neural architectures, which have evolved to self-regulate human embryogenesis and, thus, the human form.

An effective theory of adaptive self-regulation of human active inference is, therefore, an essential requirement to assure that we achieve the effective human consciousness of a PNE. The good regulator theorem of classical cybernetics proposes that a control process that fully regulates a system, like an industrial process or an organism, must constitute a model of how that system works. The model is required in order for the control process to adequately predict and, therefore, regulate, the system [70]. This theorem emphasizes that, unlike typical artificial neural networks that are operated for limited purposes under external control, an effective PNE must retain the person’s native capacity for self-regulation (the control systems of the personality).

The challenges of self-regulation and manifesting sentience have become an integral component of the literature on active inference [58]. Extending the good regulator theorem to the self-regulation of an organism, the adequately self-regulating mind must be able to model itself. Through this process of self-regulation, we could reason that a successful hybridization with the PNE, like any self-regulating system, would naturally come to model itself on the process of achieving effective self-control, thereby igniting a hybrid form of consciousness, extending across the boundary between the brain/mind and AI. Because the human brain/mind has achieved its self-regulation through elaborating primordial themes of vertebrate development, we can see that it will be important to maintain the continuity of the next stages of intelligence with the evolutionary process of self-regulation that has guided the adaptive control of active inference to this point in the evolution of intelligence.

### 4.7. Limitations

The present exercise is certainly not a practical proposal. Even as a theoretical exercise, the extent to which the assertions exceed current engineering capacities limits its value as a thought experiment. In addition, the scientific limitations of the present proposal should be recognized as well. The theory that cognition, emotion, and psychological function are identical to the process of neural development [9] is a cornerstone of our physicalist theory of mind. Even though the proposal is a decade old now, its acceptance in psychology and neuroscience is limited.

Perhaps more importantly, the extension of the theory of active inference to imply that the mind is not inherently restricted to its mortal weights, but could extend to a more general informatic implementation, may exceed even an optimistic Bayesian logic. Although we propose this optimistic interpretation in the present paper, we recognize this may exceed the bounds of what can be reasonably expected from the theory.

A specific and important limitation is that we have only vaguely characterized the methods of neuromorphic engineering that implement computational systems based on actual principles of spiking neurons. Progress in neuromorphic engineering has clarified how distributed representations, such as those emphasized in the present paper, could be modeled within populations of spiking neurons [71,72]. This work is particularly important to the goal of modeling the brain’s electrical signals with computational models of neural networks. Our limited integration of this model is an important shortcoming in our assertion that an engineering-based implementation of a PNE is feasible in principle.

Finally, the specific strategy for observing and emulating the connection weights of the personal self, in regard to the neurophysiological dynamics of memory consolidation, depends on a novel theory of the complementary roles of NREM and REM in organizing active inference. This theory is only provisionally formulated and not yet accepted by the scientific community.

### 4.8. Conclusions: Human Experience Is Computable, and the Self Is Implicit in Our Dreams

Although there are obvious and significant limitations to the present approach, our thought experiment has allowed us to propose that a high-definition AI emulation of a person’s brain/mind, and the hybridization of intelligence that is likely to result, must create not only an abstract intelligence, but the coherent experience of the self, which includes not only cognitive capacities and familiar emotional responses, but the embodied, enactive developmental memories that constitute the Bayesian self. In support of this possibility, we propose that the theory of active inference can be formulated in terms of general information theory [7], suitable for an adequate reconstruction in durable form, and it will not be restricted to describing the mortal weights of biological brains. The emulation of the mind’s structure can begin with a neuromorphic architectural model, which can then be trained (preferably by an active inference AI) to predict the person’s behavior, now readily captured in a rich digital archive, simultaneous with predicting the electrophysiological fields of the constituent neural networks.

The question then becomes how to capture the developmental memories that give the subjective substance of personal, historical experience. We propose that the solution may be to capture the information of many nights of memory consolidation, reflected in the activity of cortical electrical fields in which the new experiences of the day are negotiated to be compatible, more or less, with the historical self that has been accumulated over a lifetime. Our proposed model is that the consolidation of recent memory during NREM is balanced by the consolidation of existing historical memory (the Bayesian priors of the developmental self) during REM sleep. By monitoring the cortical electrical fields at a high resolution and, of course, being able to interpret the neurophysiological activity of the cortex in relation to its mnemonic representations, it should be feasible to reconstruct, emulate and, thus, extend the essential self in its current form, even as it takes on new capacities in the transition to metaform. Interfaced with AI, the current form of the self is not the literal history of the person’s life, but rather the essential self that is carried forward by a lifetime of consolidating experience through sleep and dreams.
